# Fine Mapping of the “black” Peel Color in Pomegranate (*Punica granatum* L.) Strongly Suggests That a Mutation in the *Anthocyanidin Reductase* (*ANR*) Gene Is Responsible for the Trait

**DOI:** 10.3389/fpls.2021.642019

**Published:** 2021-02-25

**Authors:** Taly Trainin, Rotem Harel-Beja, Irit Bar-Ya’akov, Zohar Ben-Simhon, Rami Yahalomi, Hamutal Borochov-Neori, Ron Ophir, Amir Sherman, Adi Doron-Faigenboim, Doron Holland

**Affiliations:** ^1^Unit of Fruit Tree Sciences, Institute of Plant Sciences, Agricultural Research Organization, Newe Ya’ar Research Center, Ramat Yishay, Israel; ^2^Southern Arava Research and Development, Hevel Eilot, Israel; ^3^Department of Fruit Tree Sciences, Institute of Plant Sciences, Agricultural Research Organization, Volcani Center, Rishon LeZion, Israel; ^4^Department of Vegetable and Field Crops, Institute of Plant Sciences, Agricultural Research Organization, Volcani Center, Rishon LeZion, Israel

**Keywords:** anthocyanins, pomegranate genetic variability, genetic mapping, fruit, *Punica granatum*, anthocyanidin reductase

## Abstract

Anthocyanins are important dietary and health-promoting substances present in high quantities in the peel and arils of the pomegranate (*Punica granatum* L.) fruit. Yet, there is a high variation in the content of anthocyanin among different pomegranate varieties. The ‘Black’ pomegranate variety (P.G.127-28) found in Israel contains exceptionally high levels of anthocyanins in its fruit peel which can reach up to two orders of magnitude higher content as compared to that of other pomegranate varieties’ peel anthocyanins. Biochemical analysis reveals that delphinidin is highly abundant in the peel of ‘Black’ variety. The pattern of anthocyanin accumulation in the fruit peel during fruit development of ‘Black’ variety differs from that of other pomegranates. High anthocyanin levels are maintained during all developmental stages. Moreover, the accumulation of anthocyanin in the fruit peel of ‘Black’ variety is not dependent on light. Genetic analysis of an F_2_ population segregating for the “black” phenotype reveals that it is determined by a single recessive gene. Genetic mapping of the F_2_ population using single nucleotide polymorphism (SNP) markers identified few markers tightly linked to the “black” phenotype. Recombination analysis of the F_2_ population and F_3_ populations narrowed the “black” trait to an area of 178.5 kb on the draft genome sequence of pomegranate *cv*. ‘Dabenzi.’ A putative *anthocyanidin reductase* (*ANR*) gene is located in this area. Only pomegranate varieties displaying the “black” trait carry a base pair deletion toward the end of the gene, causing a frame shift resulting in a shorter protein. We propose that this mutation in the *ANR* gene is responsible for the different anthocyanin composition and high anthocyanin levels of the “black” trait in pomegranate.

## Introduction

The pomegranate fruit is well known for its high content of health-promoting substances (Heber et al., 2006; Seeram et al., 2006). Among these are anthocyanins, which are produced in the peel and arils (the edible part of the pomegranate fruit) of the fruit (Gil et al., 1995; Hernández et al., 1999; Tzulker et al., 2007). However, there is high variation in the content and composition of anthocyanins among different pomegranate varieties (Bar-Ya’akov et al., 2019). The presence of anthocyanins in the fruit peel and arils contributes to their antioxidant activity and plays an important role in protecting the fruit from sunburns and in its attractiveness for pests (Winkel-Shirley, 2001; Koes et al., 2005). In addition, anthocyanins are the main metabolites that determine the color of the pomegranate fruit and high anthocyanin levels are a key factor in determining the economic value of the fruit. Anthocyanin level in the pomegranate fruit is developmentally regulated (Gil et al., 1995; Ben-Simhon et al., 2011; Qin et al., 2017; Yuan et al., 2017; Harel-Beja et al., 2019) and is dependent on environmental conditions such as abiotic stresses (Borochov-Neori et al., 2011; Bar-Ya’akov et al., 2019), salinity (Borochov-Neori et al., 2014), temperature and drought (Borochov-Neori et al., 2009; Holland and Bar-Ya’akov, 2014; Bar-Ya’akov et al., 2019). Numerous studies have shown that the biosynthesis of anthocyanins in maturing fruit such as apples or grapes is a light-dependent process (Mancinelli, 1985; Takos et al., 2006; Azuma et al., 2012; Jaakola, 2013).

**FIGURE 1:**
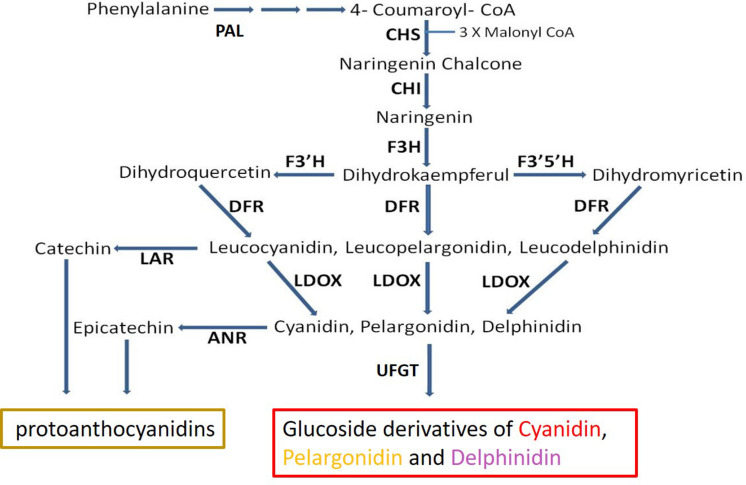
Schematic representation of the flavonoid-biosynthesis of anthocyanins and proanthocyanidins. Enzyme name abbreviations are as follows: PAL, phenylalanine ammonia-lyase; CHS, chalcone synthase; CHI, chalcone isomerase; F3H, flavanone 3-hydroxylase; F3′H, flavonoid 3′-hydroxylase; F3′5′H, flavonoid 3′,5′-hydroxylase; DFR, dihydroflavonol reductase; LDOX, leucoanthocyanidin oxidase; ANS, anthocyanidin synthase; LAR, leucoanthocyanidin reductase; ANR, anthocyanidin reductase; UFGT, UDP glucose:flavonoid 3-*O*-glucosyltransferase. Adapted from Ben-Simhon et al. (2011).

The main anthocyanins produced in pomegranate consist of the mono- and di-glycosidic forms of cyanidin, delphinidin, and pelargonidin (Gil et al., 1995; Du et al., 2006; Seeram et al., 2006). Much effort was dedicated to understanding the control of anthocyanin in plants including pomegranate (Holton and Cornish, 1995; Ben-Simhon et al., 2011; Zhao et al., 2013; Yuan et al., 2017; Harel-Beja et al., 2019). Regulation of the expression of key enzymes in the anthocyanin biosynthetic pathway via a ternary complex of MYB-bHLH-WD40 transcription factors (MBW complex) was reported for various plants: grapes (Boss et al., 1996; Cutanda-Perez et al., 2009), apples (Ban et al., 2007), pomegranate (Ben-Simhon et al., 2011), and others (Petroni and Tonelli, 2011; Albert et al., 2014). Anthocyanin level can also be regulated by further metabolism or competition on parallel pathways that utilize the same substrate (Gao et al., 2020). The proanthocyanidins (condensed tannins) pathway splits from the anthocyanin pathway via enzymes including leucoanthocyanidin reductase (LAR) and ANR (Figure 1). LAR converts leucoanthocyanidin into catechin, while ANR acts downstream to leucoanthocyanidin dioxygenase (LDOX) and converts anthocyanidins (e.g., cyanidin. pelargonidin, delphinidin) into epicatechins (Xie et al., 2003, 2004; Bogs et al., 2005). By doing so ANR can compete with the enzyme UDP-glucose: flavonoid 3-*O*-glucosyltransferase (UFGT), which converts anthocyanidin into anthocyanin (Bogs et al., 2005), and to divert the metabolism away from production of anthocyanin toward production of epicatechin (Figure 1). Overexpression of *ANR* in flower petals of tobacco results in loss of color (Xie et al., 2003; Bogs et al., 2005; Han et al., 2012). In *Arabidopsis*, a mutation in the *ANR* gene results in a colored seed coat due to the presence of anthocyanins (Xie et al., 2003).

The genes encoding for anthocyanin biosynthesis were identified in many plant species and most of them show high homology in their encoded amino acid (aa) sequences. Most of the genes known today in pomegranate were identified by homology to known functional genes in other plant species (Ben-Simhon et al., 2011, 2015; Zhao et al., 2013; Qin et al., 2017; Yuan et al., 2017). The whole pomegranate genome sequence together with transcriptomic data provided a comprehensive view of the anthocyanin pathway in pomegranate and of the expression of the genes during fruit development (Ophir et al., 2014; Qin et al., 2017; Yuan et al., 2017; Harel-Beja et al., 2019). Despite this, the biological and biochemical function of anthocyanin genes in pomegranate was shown only for very few genes. These include the *LDOX* (Ben-Simhon et al., 2015) and the *WD40* homologous genes (Ben-Simhon et al., 2011). As in other plant species, it appears that anthocyanin biosynthesis is transcriptionally regulated (Ben-Simhon et al., 2011, 2015; Zhao et al., 2013; Rouholamin et al., 2015; Qin et al., 2017; Arlotta et al., 2020).

Pronounced color differences among various pomegranate cultivars were reported (Tzulker et al., 2007; Holland et al., 2009; Bar-Ya’akov et al., 2019), suggesting high genetic variability in the regulation of pomegranate fruit color. The variation found among pomegranate varieties is in both the amount and composition of anthocyanins (Gil et al., 1995; Tzulker et al., 2007; Holland et al., 2009; Zhao et al., 2013; Harel-Beja et al., 2019). It was found that the kinetics of color appearance during fruit development is different among cultivars and that this difference is also correlated with the expression of a *MYB* gene (Ben-Simhon et al., 2011; Khaksar et al., 2015) and other regulatory genes that belong to bHLH and MYB families (Harel-Beja et al., 2019). An interesting example is the “white” pomegranate variety, which does not produce anthocyanins in any of its tissues (Ben-Simhon et al., 2015). It was shown that this phenomenon resulted from a mutation in the *LDOX* gene, which totally abolished its expression (Ben-Simhon et al., 2015). Despite the commercial and physiological importance of anthocyanin content and accumulation in pomegranate, the genetic functions responsible for most of the variability of color accumulation and production in pomegranate are not yet known.

“Black” pomegranates which are characterized by purple to black peel color are known from China in eastern Asia to western Asia (Iran, Afghanistan, Iraq, Israel) and differ from each other with respect to fruit characteristics such as taste, growth habit and dwarfism (Holland et al., 2009; Shams Ardekani et al., 2011; Özgüven et al., 2012; Zhu et al., 2015; Balli et al., 2020). In this manuscript, we focused our studies on the ‘Black’ pomegranate (P.G.127-28), which contains an exceptionally high amount of cyanidin and delphinidin mono- and di-glycosidic forms in its peel. Genetic studies have led to fine mapping of the region responsible for the “black” phenotype. These studies strongly suggest that a mutation in the *ANR* gene, situated in this region, is responsible for the “black” phenotype.

## Materials and Methods

### Plant Material

The ‘Black’ pomegranate variety P.G.127-28 was used in this research. ‘Black’ is a late season ripening variety, with a dark purple (“black”) peel color starting to accumulate in early stages of fruit development, pinkish-red arils, and a sweet taste. Other pomegranate varieties used in this study are described in Supplementary Table 1. All varieties are part of ARO’s pomegranate germplasm collection, which is located at the Newe Ya’ar Research Center^1^. The Newe Ya’ar Research Center is located in the western Yizre’el Valley, lat.32°42′N, long.35°11′E, planted on clay grumusol (vertisol) soil at an elevation of about 100 m above sea level. Yizre’el Valley is characterized by a Mediterranean subtropical climate, with an average annual rainfall of about 580 mm concentrated from November through March. The mean diurnal minimum temperature in January is 6°C, and the mean diurnal maximum temperature in July is 33°C.

A segregating F_2_ population, designated ‘Nana × Black’ (*n* = 204), was constructed from a cross between ‘Black’ (pollen donor) and ‘Nana’ seedling selection (P.G.232-243, *P. granatum* var. nana) (Harel Beja et al., 2015). Two F_3_ segregating populations were generated from two F_2_ plants as described in the “Results” Section.

**FIGURE 2:**
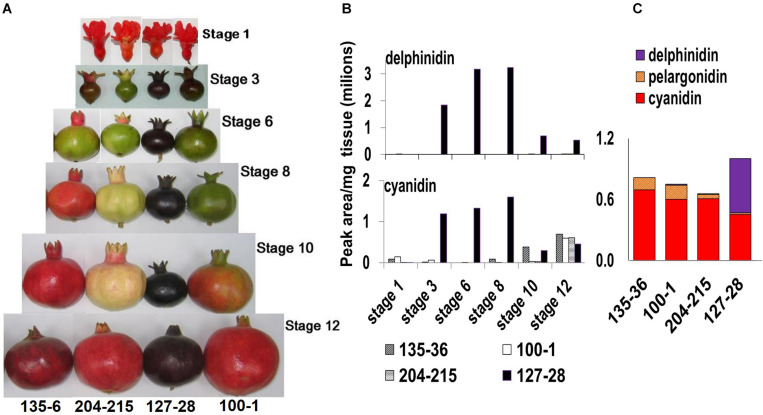
Phenotypical and chemical characterization of the ‘Black’ pomegranate compared with various “red” pomegranate varieties. **(A)** Fruits of four pomegranate varieties at six different developmental stages (Ben-Simhon et al., 2011), from flower (stage 1) to fully mature fruit (stage 12). **(B)** Total level of delphinidin and its derivatives (purple color) and of cyanidin and its derivatives (red color) in the peel during the various developmental stages of the fruit in four pomegranate varieties. **(C)** Anthocyanin composition in the peel of ripe fruit- stage 12. The varieties shown are P.G.100-1, P.G.135-36, P.G.204-215, and P.G.127-28 (‘Black’).

Fruits were collected during the years 2008–2020 at various developmental stages, from flower to fully mature fruit (Figure 2) (Ben-Simhon et al., 2011). For RNA-seq analysis fruits were collected from a very early stage after setting and before stage 3. The number of fruits from each variety for each stage was different (ten from early stages to three from stages 8–12), due to the difference in size. The fruits were photographed. For further analysis, the thinnest possible colored peel skin was removed with a sharp knife and stored at −80°C.

**FIGURE 3:**
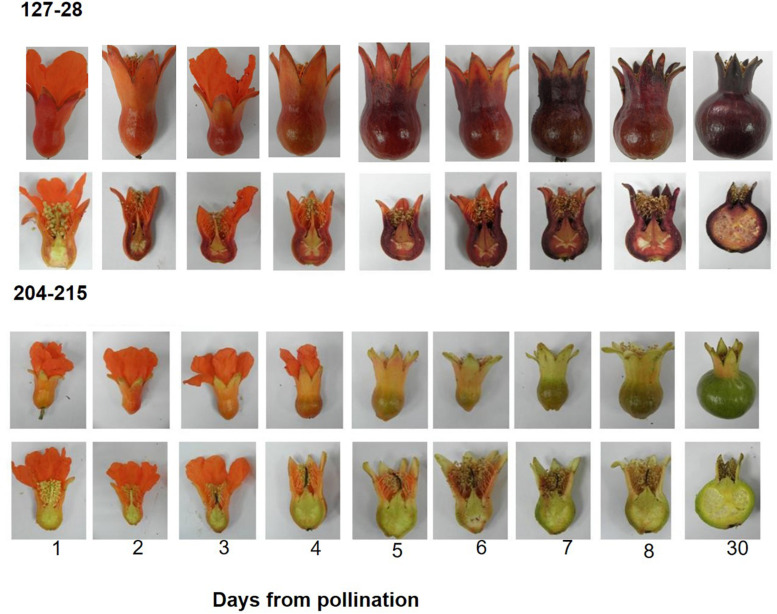
Timing of color accumulation in peel skin following hand pollination in the ‘Black’ pomegranate (P.G. 127-28) and a “red” variety (P.G.204-215). Young fruitlets were collected every day after pollination for 8 days and then 30 days after pollination (stage 2). Whole and half fruitlets are presented in order to show color development.

For determining the timing of color accumulation in summer 2014, flowers of ‘Black’ and P.G.204-215 were hand pollinated and then photographed every 24 h (Figure 3).

The plant material used for the experiment investigating light influence on anthocyanin production, which was conducted in the field, is specified in Supplementary Table 1.

### Phenotype Evaluation of Segregating Populations

Fruit peel color of the F_2_ and F_3_ populations was evaluated visually at ripening. Fruits were designated either “black” (black-purple peel color as in ‘Black’) or “red” (not black, including a largescale of pink- orange- red, pale to intense color). For quantitative analysis, three mature fruits from F_2_ siblings (*n* = 82) were sampled and used for spectroscopic analysis of total anthocyanins.

### DNA Extraction

DNA extraction was done according to Harel Beja et al. (2015) based on Porebski et al. (1997).

### Reversed Phase HPLC Analysis of Anthocyanins

Sample preparation and analysis were carried out as previously described (Ben-Simhon et al., 2011). In short, methanolic extracts of pomegranate peel tissue were analyzed using a LaChrom Merck Hitachi HPLC system coupled with a diode array detector with 3D feature (Multiwavelength Detector, Jasco MD-2010 Plus), interface (Jasco LC-Net II/ADC) and scientific software (EZChrom Elite Client/Server version 3.1.6 build 3.1.6.2433). Extract aliquots were applied to a LiChrospher 100 RP-18 column with guard column (LichroCART cartridge, Merck Millipore), and eluted with a binary mobile phase consisting of phosphoric acid (0.1% v/v, pH 2.4) and acetonitrile. Anthocyanin identification and quantification were achieved using authentic standards. For each sampling stage, extract of peel tissue pooled from four to eight flowers/fruits from three replicate trees of each pomegranate variety was analyzed.

### Spectroscopic Measurement of Total Anthocyanins

Total anthocyanin content was determined following Rabino and Mancinelli (1986). Frozen, grounded tissue (0.15 g fresh weight) was extracted in 1.5 mL of methanol containing 1% (v/v) HCl. The extract was centrifuged at 4°C, 14000 rpm for 10 min. Absorption of the extracts at wavelengths of 530 and 657 nm was determined photo metrically (CARY 50 Bio UV-visible Spectrophotometer, Agilent Technologies Inc., Santa Clara, CA, United States). When the absorption value exceeded 1.5, extracts were diluted with acidic methanol for the measurements. The anthocyanin content in the supernatant was calculated using the formula *A*530 – 0.25^∗^*A*657, allowing for the subtraction of chlorophyll interference. Results are shown as total anthocyanins per gram fresh weight. Each result is an average of three measurements.

### Tissue Culture of Fruit Cells

Calli were developed from cultured ovary mesocarp cells of the ‘Black’ variety and a “red” variety (P.G.372-383). Unfertilized hermaphroditic flowers were collected. The flowers were surface sterilized (EtOH 70% for 5 min followed by three washes in sterilized water, sodium hypochlorite 0.25% for 5 min followed by three washes in sterilized water). Then pieces of the ovary mesocarp with ovules attached were excised and placed on a regeneration medium as described in Terakami et al. (2007): MS medium (Murashige and Skoog 1962) supplemented with 0.5 μM a-naphthalene acetic acid [NAA], 5 μM N6-benzyladenine [BA], and 0.3% phytagel (Sigma-Aldrich Corp., Milwaukee, WI, United States) at pH 5.8. The plates were kept in the dark (covered with aluminum foil) for 2 weeks to encourage callus formation, and then were subjected for further experiments. Growth room conditions were 24°C and 16 h light (fluorescent light).

### Light Influence on Anthocyanin Accumulation

The effect of light on anthocyanin accumulation in pomegranate peels was studied in tissue culture of fruit cells and on whole fruits grown in the field.

#### Tissue Culture

Calli formed in the dark for 2 weeks were exposed to light. Half were kept in the dark for another 2 weeks. Then, the calli were photographed.

#### Whole Fruit

The experiment on intact fruits was conducted in the field. Fruits of three “black” peel accessions and 27 “red” peel accessions of the Newe Ya’ar pomegranate collection were covered with aluminum bags that do not allow light penetration. The fruits were covered in mid-July, 1–3 months before ripening, depending on the ripening date of each accession. The color of mature fruits from covered and uncovered controls was analyzed. Fruit color was evaluated visually and fruits were photographed. Total anthocyanin content was measured spectroscopically in peels of covered vs. uncovered mature fruits. The “black” peel and “red” peel accessions used are listed in Supplementary Table 1.

The experiment with the F_2_ population included ten “red” peel progenies and ten “black” peel progenies. For each progeny about five young fruitlets were covered with aluminum bags during May through June, and five were marked and left uncovered. Upon ripening fruits were analyzed as described above.

### Mapping the “Black” Trait in the F_2_ Population

The “black” trait was mapped to the published pomegranate genetic map (Harel Beja et al., 2015). Mapping was performed using JoinMap 3.0 software (Van Ooijen and Voorrips, 2001). The software uses Kosambi mapping functions to translate recombination frequency into map distance. Markers were grouped at a minimum LOD score of 4.0 and a recombination frequency value of 0.4.

MapQTL 5 software (Van Ooijen, 2004) was used for QTL analysis by interval mapping (IM), MQM, and for permutation analysis (1000 permutation, *p* < 0.05).

### Genotyping With Markers Close to the “Black” Trait

Genetic markers that were mapped close to the “black” peel trait were blasted to the WGS of pomegranate cv. ‘Dabenzi’ (296 Mb) reported by Qin et al. (2017) using BLASTN (Altschul et al., 1990). Six SNP markers and two SSR markers were developed within the genomic region associated with the “black” trait (Supplementary Table 2).

The F_2_ (*n* = 83) and F_3_ (*n* = 240) segregating populations were genotyped with SSR markers by fluorescence labeling using 3130 Genetic Analyzer (Applied Biosystems, Foster City, CA, United States). The PCR mixture for SSR amplification contained: 30 ng plant genomic DNA, 0.2 pmol primers, 2X Taq red master mix (Apex Bioresearch Products, Genesee Scientific Corporation, El Cajon, CA, United States) in a total volume of 20 μl. PCR conditions: 96°C for 2 min, 30 cycles of 94°C for 15 s, 55°C for 30 s and 72°C for 30 s, followed by 72°C for 45 min.

SNP markers were genotyped by sequencing PCR products with 3130 Genetic Analyzer (Applied Biosystems, Foster City, CA, United States).

### RNA-Seq of “black” vs. “red” Fruit

To further elucidate the “black” peel trait, RNA-seq analysis of fruit peels of very young fruit, i.e., after pollination and before stage 3, was performed. At this stage “black” fruits had already gained their color while “red” fruits were on the verge of becoming green (losing the red color) (Figure 2). Two varieties, ‘Black’ and ‘Nana,’ the parents of the F_2_ population, as well as twelve representatives of the F_2_ population (six with “red” peel and six with “black” peel) were selected for RNA-seq analysis. A sample consisted of the peel skin of ten fruits collected from different branches on the same tree. The tissues were crushed, immediately frozen in liquid nitrogen and kept at −80°C until further analysis. Total RNA was extracted using Norgen Plant/Fungi Total RNA Purification Kit (Norgen Biotek Corporation, Thorold, ON, Canada) including DNase treatment with RNase-free DNase I (Epicenter Biotechnologies, Madison, WI, United States) on the column, as described in the kit protocol, to remove genomic DNA. Total RNA was quantified spectrophotometrically (ND- 1000 spectrophotometer, NanoDrop Technologies, Wilmington, DE, United States), and RNA integrity was examined on a 2200 TapeStation instrument using RNA ScreenTape sample buffer and RNA ScreenTapes (Agilent Technologies, Santa Clara, CA, United States). RNA samples for RNA-seq analysis consisted of three biological replicates of ‘Black’ and ‘Nana’ (three sets of fruit taken from the same tree). Construction and sequencing of mRNA-seq libraries were performed at the Crown Genomics Institute of the Nancy and Stephen Grand Israel National Center for Personalized Medicine, Weizmann Institute of Science (G-INCPM). Briefly, mRNA-seq libraries were generated from total RNA, using the INCPM mRNA Seq protocol. The indexed libraries were pooled and subjected to sequencing on an Illumina HiSeq4000 instrument (Illumina, San Diego, CA, United States).

### Transcriptome Analysis

Raw-reads were subjected to a filtering and cleaning procedure. The SortMeRNA tool was used to filter out rRNA. Next, the FASTX Toolkit^2^ (version 0.0.13.2) was used to trim read-end nucleotides with quality scores < 30, using the FASTQ Quality Trimmer, and to remove reads with less than 70% base pairs with a quality score ≤ 30 using the FASTQ Quality Filter. Clean reads were mapped to the reference genome of *Punica granatum*^3^ using Tophat2 software v. 2.1 (Kim et al., 2013) with an average mapping rate of 95.8%. Gene abundance estimation was performed using Cufflinks v. 2.2 (Trapnell et al., 2010), combined with gene annotations^4^. Heatmap visualization was performed using R Bioconductor (Gentleman et al., 2004). Gene expression values were computed as FPKM (Fragments per kilo base per million mapped reads). Differential expression analysis was completed using the edgeR R package (Robinson et al., 2010). Genes that were more than twofold differentially expressed with false discovery-corrected statistical significance of at most 0.05 were considered differentially expressed. Venn diagrams were generated using the online tool at bioinformatics.psb.ugent.be/webtools/Venn/.

The gene sequences were used as a query term for a search of the NCBI non-redundant (nr) protein database that was carried out with the DIAMOND program (Buchfink et al., 2014). Homologous sequences were also identified by searching against the *Eucalyptus grandis* genome with the BLAST tool and an *E*-value threshold of 10^–5^. The search results were imported into Blast2GO version 4.0 for GO assignments. GO enrichment analysis was carried out using Blast2GO program based on Fisher’s Exact Test with multiple testing correction of false discovery rate (FDR). KOBAS 3.0 tool^5^ was used to detect the statistical enrichment of differential expression genes in KEGG (Kyoto Encyclopedia of Genes and Genomes) pathway and GO.

### Sequence Analysis of *ANR*

*ANR* full length transcript sequences of ‘Nana’ and ‘Black’ were cloned and sequenced. Primers used for the full-length transcribed gene were p.g.ANR F1 and p.g.ANR R1 (Supplementary Table 2). In addition, a fragment of the gene was amplified with specific primers ANR4-F4 and ANR5-R5 (Supplementary Table 2). PCR products were sequenced with 3130 Genetic Analyzer (Applied Biosystems, Foster City, CA, United States). *In silico* characterization and phylogenetic analysis of *ANR* were performed using various online applications. Virtual translation of the gene was done using the Expasy proteomics server^6^ (Artimo et al., 2012). NCBI Blastp was used to find homologous proteins and conserved domains^7^. Multiple alignment was done with MultAlin sequence alignment tool^8^ (Corpet, 1988).

### Statistical Analysis

Means, standard deviations and Wilcoxon/Kruskal–Wallis test (rank sums) analyses were conducted with the JMP program, v. 14.0 (SAS Institute Inc., Cary, NC, United States).

## Results

### The ‘Black’ Pomegranate Contains Unusual High Content of Delphinidin and Cyanidin in the Fruit Peel Skin

The ‘Black’ pomegranate variety found in Israel (P.G.127-28) is characterized by a very distinct deep-purple peel skin (“black,” Figure 2A). HPLC analysis of the anthocyanins in the peel revealed major differences between the ‘Black’ variety and three “red” varieties. Delphinidin (both of its forms, mono- and di-glycosidic) was identified at very high levels in the ‘Black’ peel skin (from here on referred to as peel). Delphinidin derivatives were responsible for the purple color. Delphinidin was not identified at all or in very minute amounts in the peel of the other three “red” varieties that were analyzed (Figures 2B,C). The ‘Black’ peel also accumulated a high level of cyanidin derivatives responsible for the red color (Figures 2B, C). The levels of cyanidin in the ‘Black’ fruit peel can reach up to two orders of magnitude as compared to other pomegranates’ peel anthocyanin content (for example, at stage 8, cyanidin level was 1,598,822 peak area/mg tissue in ‘Black’ and only 2,723 in the “red’ variety P.G.100-1). Moreover, the accumulation of anthocyanins in the ‘Black’ variety started already in the early stage 3 of fruit development (stages as described in Ben-Simhon et al., 2011) contrary to the “red” accessions analyzed in this study that did not accumulate anthocyanins at this stage. As can be seen in Figure 2A, ‘Black’ is “black” from stage 3 on, while the other varieties lose anthocyanins at this stage and regain color again later on in fruit development. The levels of delphinidin and cyanidin were reduced in the ‘Black’ variety at later stages, but the color of the fruit was not diluted visually. Still, total anthocyanin was 1.2- to 1.5-fold higher in the ‘Black’ than in the examined “red” varieties at stage 12 (Figure 2C). Total anthocyanins in ‘Black’ ripe fruits consisted of 54% delphinidin, 45% cyanidin and only 2% pelargonidin. In the “red” fruit the major anthocyanin is cyanidin, constituting 80–90% of total anthocyanins. Pelargonidin constitutes 7–18% and delphinidin is either absent or found in very small amounts (0–2%).

It seems that the unique accumulation of delphinidin together with the high levels of cyanidin and delphinidin anthocyanins accumulating from early stages of fruit development is responsible for the special “black” phenotype.

### The ‘Black’ Pomegranate Starts Accumulating Color Right After Pollination

A daily follow up of color appearance since flower pollination during the development into young fruit (30 days, stage 2) clearly demonstrated that “black” color accumulation begins very early in fruit development (Figure 3). The “black” color already started to accumulate in the sepals of the ‘Black’ variety 1 day following pollination. From this stage on, the “black” color was maintained throughout fruit development. In the “red” variety, however, a few days after pollination the red color of the sepals faded and was replaced by the green color of the chlorophyll. “red” varieties accumulated anthocyanins at later stages of fruit development toward ripening (Figure 2), the rate and precise timing of accumulation varied between the different cultivars. Chlorophyll and carotenoid contents in peels of the ‘Black’ variety did not vary from the “red” accessions (data not shown). The difference in color can thus be attributed to anthocyanin content. The time of color appearance in ‘Black’ is another unique trait that differentiates it from other varieties.

### Color Accumulation in the ‘Black’ Pomegranate Does Not Appear to Depend on Light

**FIGURE 4:**
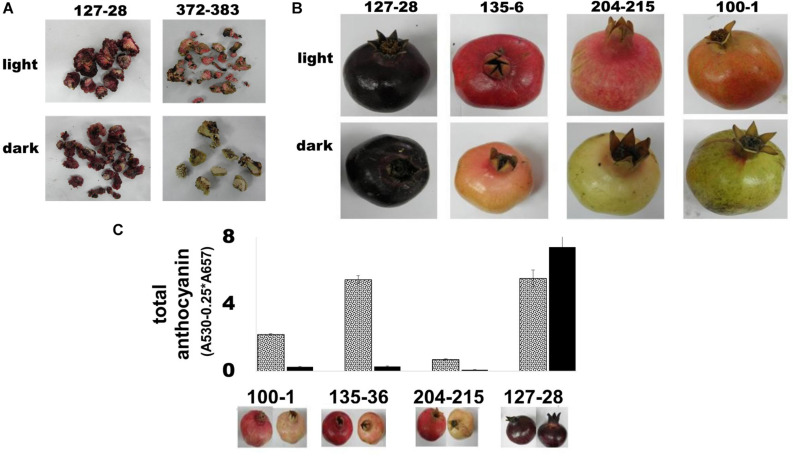
Color accumulation in fruit and tissue culture of the ‘Black’ pomegranate in the absence of light. **(A)** Light influence on color development in calli derived from the ovary mesocarp of flowers from the ‘Black’ pomegranate and the “red” variety P.G.372-383. The calli were photographed after 2 weeks in complete darkness **(B)** Light influence on peel color in intact fruit. Fruits at stage 6 were covered with aluminum bags, which prevented any exposure to light. At ripening, covered fruits were compared to uncovered fruits. The figure shows three representative “red” varieties in addition to the ‘Black’ pomegranate. **(C)** Total amount of anthocyanin in the peel of fully ripe fruit grown in the dark (full black column) was compared with that of control uncovered fruit exposed to light (gray dotted column). Under each column there is an image that represents the uncovered fruit (left) and covered fruit (right).

The effect of light on anthocyanin accumulation in pomegranate peels was studied in tissue culture of fruit cells and on whole fruits grown in the field. In the first experiment, calli developed from cultured ovary mesocarp cells taken from flowers of the ‘Black’ variety and a “red” accession P.G.372-383 were compared in response to light deprivation. Plates containing 4 weeks-old cultured calli were covered with aluminum foil to avoid light penetration. After 2 weeks the plates were transferred to regular light conditions. As can be seen in Figure 4A, the calli of the “red” variety lost their anthocyanins in the dark, while the calli originating from the ‘Black’ pomegranate remained colored. The “red” pomegranate calli accumulated red color as soon as 1 day after returning to light.

The second experiment was conducted in the field on intact fruits. Fruits of the ‘Black’ pomegranate, as well as 4 “black” peel varieties and 27 “red” varieties of the Newe Ya’ar pomegranate collection (Supplementary Table 1) were covered with aluminum bags that do not allow light penetration. The fruits were covered at stage 6, before red color developed; however, “black” fruits were already colored at that stage. At ripening “black” pomegranate fruits retained their deep purple color regardless of light exposure, while the “red” varieties lost their color in the dark (Figure 4B).

The effect of light deprivation was also evaluated quantitatively using spectrophotometric analysis of total anthocyanins in peels of covered vs. uncovered mature fruits. As shown in Figure 4C, total anthocyanins in the fruit peel of “red” varieties decreased up to 20-fold in the dark (P.G. 135-36; 5.46 in the light vs. 0.28 in the dark). By contrast, total anthocyanins in the fruit peel of ‘Black’ pomegranate increased in covered fruit.

Interestingly, insensitivity to light appears to be specific to color accumulation in the fruit as the etiolation response of seedlings of the ‘Black’ pomegranate was similar to that of the “red” pomegranates.

It seems that the ‘Black’ pomegranate lost its sensitivity to light with respect to color development in the fruit peel.

### Inheritance of the “black” Phenotype in Pomegranate

**FIGURE 5:**
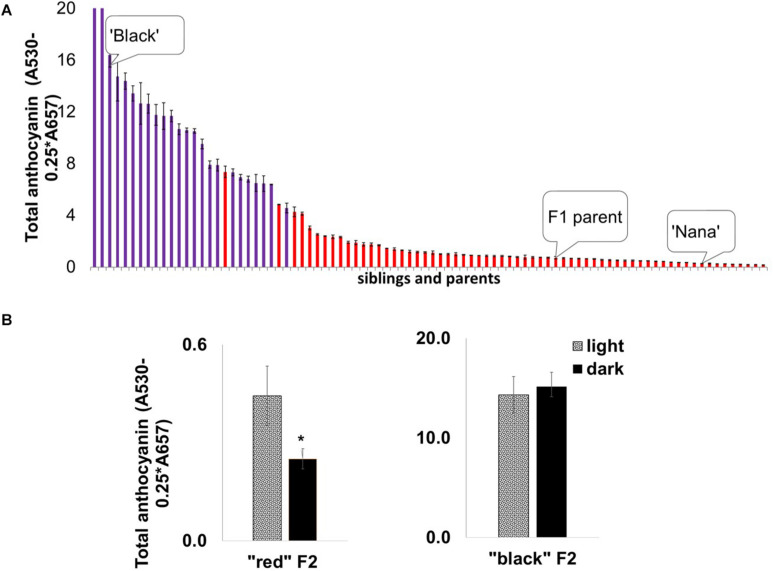
Segregation of anthocyanin in mature fruit peels and the effect of depletion of light on peel color development in the ‘Nana × Black’ F_2_ population. **(A)** Distribution of total anthocyanin amount in the F_2_ progeny (*n* = 82) for “black” peel (purple columns) and “red” peel (red columns). The parents, ‘Black’ and ‘Nana’ and the F_1_ offspring whose self-pollination created this F_2_ population are marked. Each column represented in the graph is an average of three extracts ± standard error. **(B)** Effect of light deprivation on “red” and “black” representatives from the F_2_ population: Total amount of anthocyanin in the peel of fruits developed in the dark (covered) compared with the control (light, uncovered fruits). Asterisk indicates a statistically significant difference (*p* < 0.05) analyzed by Wilcoxon/Kruskal–Wallis test (rank sums) and by non-parametric comparisons probability (*F* = 0.03).

We undertook a mapping approach to identify the mode of inheritance of the “black” phenotype and the gene(s) responsible for it. Initially, mapping studies were based on an F_2_ ‘Nana × Black’ population originating from a cross between the ‘Black’ pomegranate and the “red” variety ‘Nana’ (P.G.232-243). This population was used earlier to construct a genetic map of pomegranate and for QTL mapping of several important traits (Harel Beja et al., 2015). The F_2_ population (204 siblings in total) segregated for the “black” and “red” phenotypes as follows: 23% (47) of the siblings showed a “black” phenotype and 77% (157) showed a “red” phenotype. These results together with the results of a ‘Black’ self-population in which all the siblings (*n* = 64) were “black,” lead to the conclusion that the “black” phenotype is controlled by a single gene showing a Mendelian inheritance ratio of one gene (1:3 ratio). The peel of the “black” siblings contained a higher level of total anthocyanins compared to the “red” peel siblings, beside one “red” with very high anthocyanin content (Figure 5A).

Interestingly, the insensitivity of the “black” fruit color to light deprivation co-segregated with the “black” phenotype in the F_2_ population. Statistical analysis (Figure 5B) showed no significant difference in total anthocyanins in covered vs. uncovered fruit in the “black” siblings. Among the “red” siblings, the covered fruits had a significantly (*p* = 0.0246) lower content of total anthocyanins, analyzed by the Wilcoxon/Kruskal–Wallis test (rank sums) and by non-parametric comparisons probability (*F* = 0.03). The association between peel color and the response to light strongly suggests that the insensitivity to light is controlled by the same gene that controls the “black” phenotype.

**FIGURE 6:**
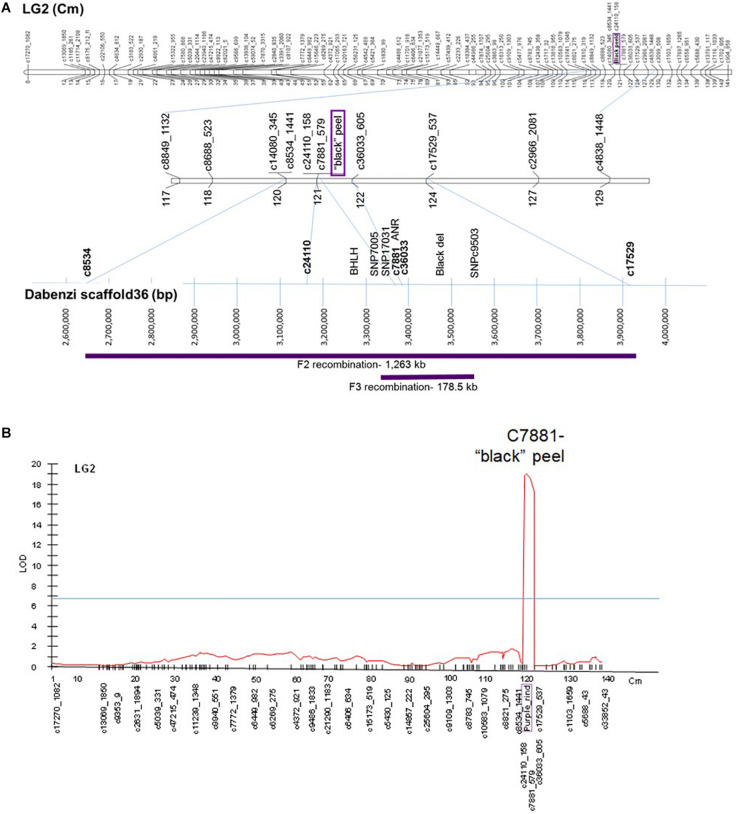
Mapping the “black” trait in pomegranate. **(A)** On top - The “black” trait (in purple) was mapped to LG2 on the published ‘Nana × Black’ F_2_ genetic map (Harel Beja et al., 2015). At the bottom - Positioning of the mapped genetic markers and additional markers on the published sequenced genome (‘Dabenzi’ scaffold36; Qin et al., 2017). The regions on the genome corresponding to the F_2_ and F_3_ recombinations are in purple. **(B)** QTL analysis (MQM) of total anthocyanins for the F_2_ progeny at LG2.

The “black” trait was mapped to LG2 on the published ‘Nana × Black’ F_2_ genetic map (Harel Beja et al., 2015) based on SNP genetic markers described in Ophir et al. (2014) (Figure 6A). Total anthocyanins content was mapped as a major QTL in the same area on LG2 (on the region of marker c7881) with a LOD score of 19 (Figure 6B). Minor QTLs were analyzed by MQM in LG3, LG6, LG9 and LG10 (Supplementary Table 3).

*F3’5’H*, the gene responsible for synthesis of delphinidin, the purple anthocyanin color that characterizes the peel of the “black” phenotype (Figure 1), was initially considered as a likely site of the “black” mutation. However, it did not map to any of the above QTLs in the F_2_ population. *F3’5’H* (Supplementary Table 2) was mapped to LG7 on the ‘Nana × Black’ F_2_ genetic map (Harel Beja et al., 2015). Therefore, it is not the gene that controls the “black” phenotype.

The sequence of genetic markers that were mapped close to the “black” peel trait was blasted using BLASTN on the WGS of pomegranate cv. ‘Dabenzi’ (296 Mb; Qin et al., 2017). This approach revealed a region of 1,263 kb on scaffold36 (MTKT01002534.1) that matched the region between the mapping markers bordering the trait (c8534-c17529, Figure 6A). This region contains 172 genes. Among them there are two annotated genes known to be involved in anthocyanin biosynthesis. One of the genes is a transcription factor *bHLH (CDL15_Pgr017019)* and the second gene is *ANR* (*CDL15_Pgr01703*2; Figure 1). bHLH type proteins are known to be part of the complex that regulates the anthocyanin biosynthetic pathway (Petroni and Tonelli, 2011). ANR acts in the anthocyanin pathway, diverting anthocyanins to epicatechin (proanthocyanidins; Figure 1). For each of these genes an SNP and/or an SSR molecular marker was created (Supplementary Table 2).

In order to fine map the “black” trait in the c8534-c17529 region, and to study which of the candidate genes is associated to the trait, advanced F_3_ populations were constructed (Table 1). Within 76 plants of the mapping F_2_ population, there were seven recombination events at the associated c8534-c17529 region. However, to distinguish between the candidate genes, two plants that showed recombination between *ANR* and *bHLH* markers were selected. These two plants were selected for self-propagation to create F_3_ populations that would show recombination within the “black” genomic region. One of the F_2_ plants was heterozygous for the *ANR* marker and homozygous for the “red” allele of the *bHLH* marker (population 1). The other F_2_ plant was the other way around, i.e., homozygous for the “red” allele of the *ANR* marker and heterozygous for the *bHLH* marker (population 2). We screened 120 plantlets of F_3_ population 1 with the *ANR* marker. Homozygous recombinant plants that were homozygous for the “black” allele of *ANR* and homozygous for the “red” allele of *bHLH* were planted in the orchard. 120 plantlets of F_3_ population 2 were screened with the *bHLH* marker. Plants that were homozygous for the “red” allele of *ANR* and homozygous for the “black” allele of *bHLH* were planted in the orchard (Table 1). Therefore, there are two F_3_ populations that differ at the “black” region genotype. Fruit peel color was described as “black” or “red” (Table 1). Twelve plants of F_3_ population 1, that were homozygous for the “black” allele of *ANR* and homozygous for the “red” allele of *bHLH* had a “black” peel. None of the plants of this genotype had a “red” peel. On the other hand, all the seven plants of F_3_ population 2 that were homozygous for the “red” allele of *ANR* and homozygous for the “black” allele of *bHLH* had “red” peel (Table 1). The recombination data (Table 2) clearly indicates that the “black” phenotype is 100% linked to the *ANR* gene but not to the *bHLH*, suggesting that the *ANR* gene might be the gene that confers the “black” phenotype. Nevertheless, other traits such as plant height and fruit size varied among the F_3_ plants. Six additional DNA markers were developed between the *bHLH* and *ANR* markers (Supplementary Table 2). Genotype analysis of the F_3_ plants from both populations with those markers showed additional 23 recombinations and reduced the site of the gene to a region of 178.5 kb on the draft pomegranate genome of ‘Dabenzi’ (Figure 6A and Table 2). This region contains 27 genes based on the sequenced pomegranate genomes (Qin et al., 2017; Yuan et al., 2017; Luo et al., 2020) and annotated genes published by Ophir et al. (2014) (Supplementary Table 4). Among those genes, *ANR* is the best candidate since it is known to function at the flavonoid pathway. Furthermore, it was differentially expressed between ‘Black’ and ‘Nana’ as is explained below.

**TABLE 1:** The allelic composition of *ANR* and *bHLH* homozygous markers and the matching peel color in two F_3_ populations.

**F_3_ population**	***ANR* marker homozygous for “black” allele**	***bHL*H marker homozygous for “black” allele**	**Peel color**
F_3_ population 1	12	0	“black”
F_3_ population 2	0	7	“red”

**TABLE 2:** Matching for F_2_ and F_3_ populations between the “black” trait and neighboring markers positioned on ‘Dabenzi’ scaffold 36 (Qin et al., 2017) sequence.

**Position on Dabenzi scaffold 36**	**2655494**	**3162458**	**3274725**	**3327005**	**3348965**	**3360974**	**3389282**	**3392436**	**3407657**	**3505550**	**3918578**

**Marker**	**c8534**	**c24110**	**bHLH**	**7005**	**17031**	**ANR**	**c7881**	**c36033**	**black del**	**c9503**	**c17529**
F_2_ progeny	95	100	100	nd	nd	100	100	100	nd	nd	95
F_3_ progeny*	nd	nd	0	0	100	100	100	100	100	91.7	91.7

The “black” trait area for each population is highlighted in purple. The distances on ‘Dabenzi’ scaffold 36 between F_2_ and F_3_ recombinations are 1,263 kb and 178.5 kb, respectively. *F3 population1. nd, not determined. Values are expressed as percentage.

### Comparative Expression Analysis of Anthocyanin Related Genes in Fruit Peels of ‘Black’ and ‘Nana’ Fruits

**FIGURE 7:**
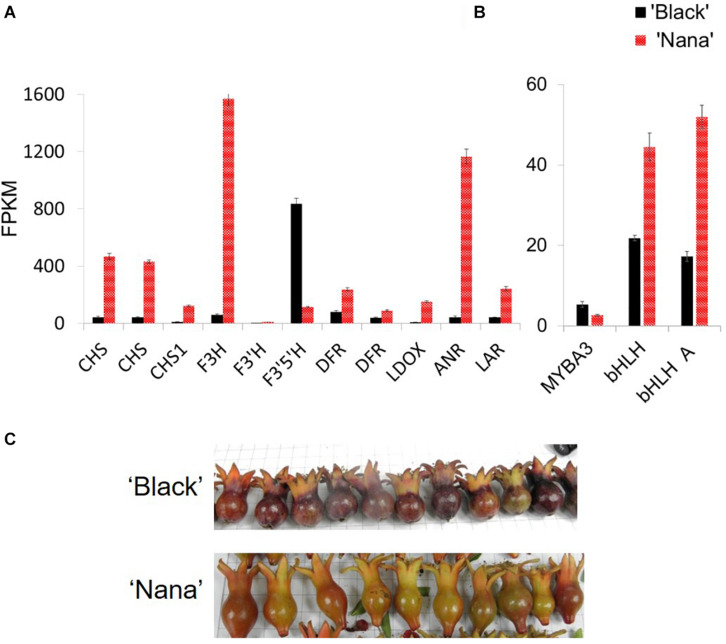
Differential expression of genes from the anthocyanin pathway in ‘Black’ and ‘Nana’ young fruitlets. Peels were collected from fruits of ‘Black’ (black columns) and ‘Nana’ (red columns). Fruitlets were collected a few days after pollination. Expression analysis is explained in detail in the Materials and Methods section. **(A)** Genes corresponding to anthocyanin pathway. Gene names according to Qin et al. (2017): left to right: CDL15_Pgr005566 (CHS), CDL15_Pgr025723 (CHS), DL15_Pgr026373 (CHS1), CDL15_Pgr020918 (F3H), CDL15_Pgr008828 (F3’H), CDL15_Pgr026644 (F3’5’H), CDL15_Pgr021400 (DFR), CDL15_Pgr021400 (DFR), CDL15_Pgr017842 (LDOX), CDL15_Pgr017032 (ANR), CDL15_Pgr024128 (LAR). **(B)** Regulatory elements. From left to right- MYBA3 (JF747151), bHLH (CDL15_Pgr025), bHLH (JF747152). **(C)** Fruitlets analyzed showing the observed peel color at this stage. FPKM, fragments per kilo base per million mapped reads.

Expression analysis was performed in order to explore the differences in the expression of the anthocyanin pathway-related genes between ‘Black’ and ‘Nana.’ For this purpose, RNA was extracted from fruit peels of fruitlets harvested before stage 3 (Figure 7C). At this stage, ‘Black’ fruits had already gained their color while ‘Nana’ fruits were on the verge of becoming green (losing the red color). Differences in expression were observed for structural genes of the anthocyanin pathway, including: *chalcone synthase* (*CHS*), *chalcone isomerase* (*CHI*), *flavanone 3-hydroxylase* (*F3H*), *dihydroflavonol reductase* (*DFR*), *flavonoid 3’5’-hydroxylase* (*F3’5’H*), *flavonoid 3’-hydroxylase* (*F3’H*), *LDOX*, *ANR* and *LAR* (Figure 7A and Figure 1). Surprisingly, the expression of these genes was higher in the peel of ‘Nana’ as compared to ‘Black,’ except for the *F3’5’H* gene.

Differential expression levels were observed for several *MYB* and *bHLH* genes (Figure 7B). These transcription factors are associated with anthocyanin biosynthesis and metabolism. Nevertheless, none of these genes was mapped to the “black” 178.5 kb region. Moreover, when the expression of the above structural or regulatory genes was analyzed at the same stage of development on fruit bulks from representative “black” or “red” fruits collected from the F_2_ population, the linkage between the expression of a specific gene and the “black” trait was lost (Supplementary Figure 1).

There were differences in the expression level of 3,799 genes between the ‘Black’ peels and the ‘Nana’ peels. However, out of the 27 genes found in the 178.5 region on scaffold36, 23 genes were found to be transcribed in both the ‘Black’ pomegranate and the ‘Nana’ peels with no dramatic difference in gene expression except for the *ANR* gene (Supplementary Table 4). When the expression of these genes was compared between “red” and “black” representatives of the F_2_ population, no correlation was seen except for the *ANR* gene, though the difference was less dramatic.

### A Single Mutation in the *ANR* Gene Could Be Responsible for the “black” Phenotype

**FIGURE 8:**
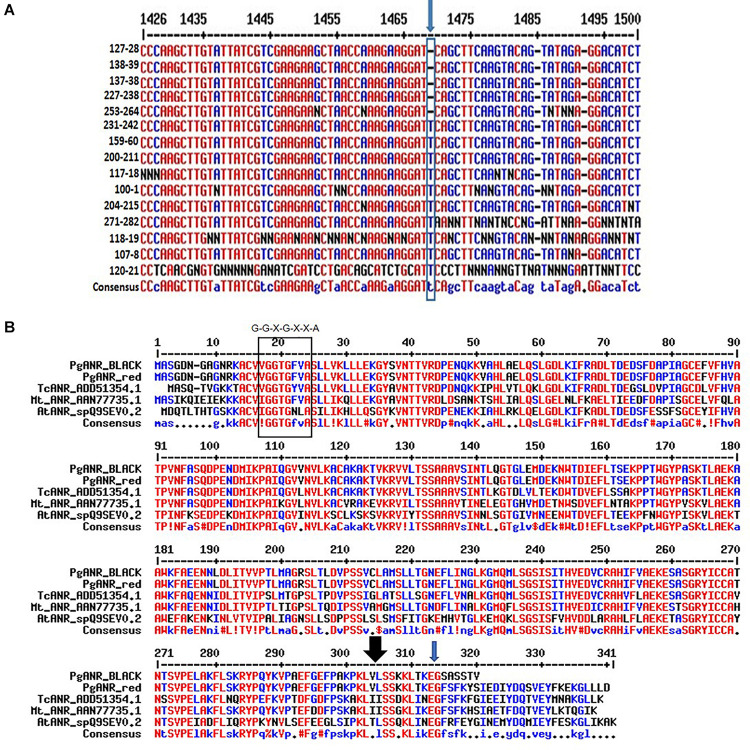
Characterization of the *ANR* mutation. **(A)** The site and nature of the *ANR* mutation in the DNA sequence corresponding to the *ANR* gene. Multiple alignment of partial nucleotide sequence showing the mutation (blue arrow). Details of varieties shown are in Supplementary Table 1. **(B)** Multiple alignment of the deduced pomegranate proteins corresponding to ANR. The rectangle at the N terminus of the protein shows the conserved Rossmann dinucleotide-binding domain amino-termini (G-G-X-G-X-X-A). The black arrow at the C terminus of the protein shows the area of a putative substrate binding site. The ANR proteins shown in the alignment are- *Arabidopsis thaliana* AtANR_spQ9SEV, *Medicago truncatula* MtANR_AAN77735.1, *Gossypium hirsutum* GhANR_ABM64802.1 and *Glycine max* G.max_ANR2_NP_001243072.1. http://multalin.toulouse.inra.fr/multalin.

Comparative genomic sequencing of the putative *ANR* gene of “red” peel and “black” peel varieties (Figure 8) revealed a single base deletion unique to “black” peel varieties. This mutation co-segregates (100%) with the “black” phenotype in the F_2_ population and among all “black” varieties in the Newe Ya’ar pomegranate collection (Figure 8A and Table 3). The single base deletion creates a (nonsense) mutation toward the end of the gene, thereby shortening the deduced protein by 25 aa (Figures 8A,B) and could potentially inactivate or reduce the activity of the ANR protein.

**TABLE 3:** The ANR mutation is unique to the “black” varieties in the Newe Ya’ar pomegranate collection.

**Pomegranate variety**	**Peel color**	***ANR* mutation**
100-1	Red	No
118-19	Red	No
120-21	Red	No
271-282	Red	No
107-8	Red	No
117-18	Red	No
200-211	Red	No
204-215	Red	No
231-242	Red	No
159-60	Red	No
127-28 ‘Black’	Black	Yes
137-38	Black	Yes
138-39	Black	Yes
227-238	Black	Yes
253-264	Black	Yes

*Only part of the red varieties analyzed are shown. All “black” varieties are shown.*

## Discussion

Pomegranate varieties characterized by “black” fruits are known from central and western Asia (Holland et al., 2009; Shams Ardekani et al., 2011; Özgüven et al., 2012; Zhu et al., 2015). Some work has been done concerning anthocyanin or flavonoid levels and composition in the peels of these “black” pomegranates (Shams Ardekani et al., 2011; Zhao et al., 2013; Zhu et al., 2015; Balli et al., 2020), as well as their health benefits (Dana et al., 2015). In agreement with our findings for the Israeli ‘Black’ pomegranate, the Persian ‘Black pomegranate’ peel has higher flavonoid content then other pomegranate cultivars (Shams Ardekani et al., 2011). The Chinese “black” peel variety ‘Moshilio’ was shown to have high levels of delphinidin and cyanidin in the peel compared to other pomegranates (Zhao et al., 2013; Zhu et al., 2015). This study undertook a genetic approach to identify the genetic components that are responsible for the “black” trait. This remarkable feature is characterized by accumulation of delphinidin together with unusual high levels of cyanidin and delphinidin in the fruit peel that accumulate from early stages of fruit development. By establishing and mapping an F_2_ population originated by a crossbred with a pomegranate variety that carries the “black” phenotype, it was possible to demonstrate that this phenotype is controlled mainly by a single recessive gene located at LG2 according to the pomegranate genetic map (Harel Beja et al., 2015). The associated region was placed on the pomegranate genomic sequence (‘Dabenzi’ scaffold36 2,655,494–3,900,000; Qin et al., 2017). Two genes positioned at that region (3,360,974 and 3,274,725 on the ‘Dabenzi’ scaffold36), *ANR* and *bHLH*, respectively, were annotated as genes that encode proteins that might function in the anthocyanin biosynthetic pathway. Fine mapping based on two additional F_3_ populations generated from two different heterozygotic progenies of the F_2_ population enabled us to reduce the “black” trait to a region of 178.5 kb. In addition, segregation analysis of the two F_3_ populations demonstrated that the “black” phenotype segregated with the *ANR* “Black” genotype, hence excluding the *bHLH* as a possible cause for the trait and strongly suggesting *ANR* as the responsible gene. This assumption was further strengthened by surveying 100 genotypes of our pomegranate collection (Holland et al., 2009). The collection includes five different “black” varieties that differ with respect to taste, fruit size, time of ripening and size of the trees. Interestingly, only pomegranate accessions carrying the “black” phenotypes have a deletion of a single T nucleotide which deduces a shorter ANR protein (Figure 8). Thus, the single T deletion is in complete linkage with the “black” phenotype in the F_2_ population, in two F_3_ populations and within the pomegranate collection. Finally, expression analysis performed on fruit peel at an early developmental stage when “black” color accumulation begins, demonstrated that the *ANR* gene expression was significantly reduced in the ‘Black’ pomegranate as compared to the “red” pomegranate ‘Nana.’ This significant change in gene expression was only seen for the *ANR*, while the other 27 genes that are present within the 178.5 kb associated region did not show significant change in their expression between ‘Black’ and ‘Nana’ or between “red” and “black” phenotypes in the F2 population.

We do not exclude the possibility that other genes in the mapped area could be the genes responsible for the “black” phenotype. There are non-synonymous mutations in other genes in the mapped region (Supplementary Table 5). However, we strongly suggest that the mutation in the *AN*R gene is responsible for the “black” phenotype for the following reasons: (1) *ANR* belongs to the anthocyanin pathway and its inactivity can explain anthocyanin accumulation. (2) Expression data, which clearly show bold differences in its expression between ‘Black’ and ‘Nana’ (a “red” pomegranate). (3) The genotyping data is consistent with the phenotype in the collection and populations. (4) There is a clear non-synonymous mutation in the *ANR* gene (Figure 8) which shows a difference between all “black” and non-black (“red”) phenotypes that were analyzed. (5) There are non-synonymous mutations in other genes in the mapped region, but they are either expressed in a very low level or show a similar expression level between “black” and “red’ phenotypes.

We hypothesize that the mutation in the *ANR* gene shortened the ANR enzyme (Figure 8B), and as a result affected its activity. ANR belongs to a group of flavonoid reductases of the extended SDR-type (NCBI, Blastp, conserved domains: Lu et al., 2020). Extended SDRs have a C-terminal extension of approximately 100 aa that is less conserved (Moummou et al., 2012; Yonekura-Sakakibara et al., 2019). Moreover, a substrate binding domain is located in the C-terminal region, which determines specificity (Petit et al., 2007). The pomegranate ANR protein is 339 aa long, in accordance with other known ANRs (Figure 8B). One of the putative substrate binding sites is at the 304 aa position (lysine 304). Interestingly, the “black” ANR is mutated at the C terminal at aa 314. The mutation causes a frameshift that shortens the protein to a predicted open reading frame encoding 319 aa. The predicted changes at the C terminal of the protein may cause different folding due to aa change or shortening of the protein, and as such may affect the binding of the substrate and the activity of the enzyme.

ANR competes for the same substrate with glycosyltransferase enzymes. While glycosyltransferases convert anthocyanidins into their mono- and di-glycosylated colored forms, ANR diverts the biosynthetic activity toward the production of proanthocyanidins, which are colorless (Xie et al., 2003; Kitamura et al., 2016). Hence, the *ANR* mutation could affect color production by reducing competition over anthocyanidins formation, resulting in overproduction of anthocyanin (Figure 1). It was shown that overexpression of *ANR* negatively affected color production in transgenic plants as *Vitis vinifera* (Bogs et al., 2005), *Theobroma cacao* (Liu et al., 2013), *Malus domestica* (Han et al., 2012) and *Fragaria chiloensis* (Salvatierra et al., 2013). The “black” phenotype in pomegranate is the first natural mutation in *ANR* published, suggesting a novel regulatory pathway that controls color production in fruit by partially or completely blocking a competing pathway. Mutations that cause color accumulation by blocking successive biosynthetic pathways were reported in the carotenoid pathway in fruit of the Cucurbitaceae family (Feder et al., 2015; Chayut et al., 2017).

Genes annotated as related to the anthocyanin pathway were expressed at a much lower level in ‘Black’ pomegranate as compared to the “red” pomegranate ‘Nana’ (Figure 7). These differences in expression level were not found in fruit bulks from representative “black” or “red” fruits of the F_2_ population (Supplementary Figure 1). This may reflect a feedback mechanism whereby high anthocyanin accumulation in the ‘Black’ pomegranate generates a negative feedback mechanism which reduces transcription of most of the anthocyanin pathway. We found one exception to this, which is the *F3’5’H* gene (Figure 7A.). Intriguingly, this gene is responsible for delphinidin biosynthesis (Tanaka and Brugliera, 2013). Nevertheless, the *F3’5’H* gene was not mapped with the “black” trait, and therefore it is not the gene controlling the “black” phenotype.

The reduction of delphinidin and cyanidin levels toward ripening (stage 12; Figure 2B) can be explained as a dilution effect caused by low anthocyanin biosynthesis accompanied by a dramatic increase in the size of the fruit (Figure 2A). In addition, former experiments indicate that the expression of structural genes involved in the anthocyanin biosynthetic pathway are very low at ripening stage of the ‘Black’ pomegranate (Ben-Simhon, 2015).

The specific high accumulation of cyanidin and delphinidin in the fruit peel and not in arils of the “black” phenotype is of interest. Since we found only one single gene encoding for ANR, we propose that the anthocyanin pathway in the arils and peels could be controlled differently. This assumption is corroborated by the fact that there is no correlation between the outer skin color of the rind and the color of the arils (Holland et al., 2009). For example, in the arils, the biosynthetic pathway that diverts anthocyanins to proanthocyanidins may not be active and therefore a mutation in *ANR* does not influence color accumulation in the arils.

Another aspect of the “black” phenotype is the insensitivity of color accumulation in response to light exposure. Numerous studies have shown that the biosynthesis of anthocyanins in maturing fruit such as apples or grapes is a light-dependent process (Takos et al., 2006; Azuma et al., 2012). As indicated in Figure 4, color accumulation in the calli and peel of ‘Black’ pomegranate was not affected by light deprivation. Light-independence was specific to color accumulation in the fruit and was not reflected in other light-dependent responses such as in etiolated germinating seedlings. Thus, elongated hypocotyls, shortened roots, and small, closed cotyledons were evident in ‘Black’ self-seedlings as well as in “red” self-seedlings when exposed to darkness. We have demonstrated by genetics that light-independence is linked to the “black” phenotype (Figure 5B). How can a mutation in *ANR* explain such a phenotype? Within the genomic associated region there may be an additional genetic component that controls sensitivity to light and due to its close proximity to the *ANR* gene on the pomegranate genome they co-segregate in the population. Perhaps this gene is the *bHLH* gene which was found in the mapped area (Figure 6A). This gene has a mutation that is unique to the “black” phenotype in F_2_ siblings as well as in the pomegranate collection. However, as discussed before, we found recombinants in the F_3_ populations which show that the *bHLH* gene does not fully segregate with the “black” phenotype. Hence, there is a possibility that the “black” phenotype could be a combination of two or more gene mutations which are closely linked to each other. Light-independence of fruit development, in addition to color, is important for the protection of the fruit and influences its commercial value. Developing fruit color without dependence on light is a most desirable trait that could increase the quality of fruit and enable colored fruit production under light-limiting conditions (e.g., in the shaded part of the tree or under nets). Therefore, deciphering the “black” mutation is of high importance in finding new methodologies for breeding and manipulating color accumulation in fruits.

## Data Availability Statement

Data of this project have been deposited with links to BioProject accession number PRJNA694423 in The National Center for Biotechnology Information (NCBI). BioSamples accession numbers: SAMN17522954, SAMN17523170, SAMN17523467, SAMN17523758 for ‘Black,’ ‘Nana,’ “red” peel bulks and “black” peel bulks, respectively. The Raw data from the Illumina sequencing have been deposited in NCBI Sequence Read Archive (SRA) under accession numbers: SRR13530002; SRR13530001; SRR13529998; SRR13529997; SRR13529996; SRR13529995; SRR13529994; SRR13529993; SRR13529992; SRR13529991; SRR13530000; SRR13529999.

## Author Contributions

TT performed the experiments, analyzed the study, and wrote the manuscript. RH-B performed the experiments and analyzed the study. IB-Y, ZB-S, and RY performed the experiments. HB-N performed chemical analyses. RO, AS, and AD-F contributed to bioinformatics. DH designed and conducted the research. All authors contributed to the article and approved the submitted version.

## Conflict of Interest

The authors declare that the research was conducted in the absence of any commercial or financial relationships that could be construed as a potential conflict of interest.

## Footnotes

1 http://igb.agri.gov.il/main/index.pl?page=22

2 http://hannonlab.cshl.edu/fastx_toolkit/index.html

3 https://www.ncbi.nlm.nih.gov/assembly/GCA_002201585.1

4 http://peppergenome.snu.ac.kr/download.php

5 http://kobas.cbi.pku.edu.cn/kobas3/?t=1

6 http://www.expasy.org

7 https://blast.ncbi.nlm.nih.gov/Blast.cgi

8 http://multalin.toulouse.inra.fr/multalin

## Abbreviations

HPLC: high performance liquid chromatography
PG: *Punica granatum*
ANR: anthocyanidin reductase
SNP: single nucleotide polymorphism
SSR: simple-sequence repeats
aa: amino acids
MQM: multiple QTL model
WGS: whole genome shotgun sequence
GO: gene ontology
LG2: linkage group 2.
