# Supplementary material for: Fine Mapping of the “black” Peel Color in Pomegranate (Punica granatum L.) Strongly Suggests That a Mutation in the Anthocyanidin Reductase (ANR) Gene Is Responsible for the Trait
Source: Front Plant Sci. 2021 Feb 25;12:642019. doi: 10.3389/fpls.2021.642019 (PMC7947214; doi:10.3389/fpls.2021.642019)
Supplement: Supplementary file 1 [file Image_1.pdf]

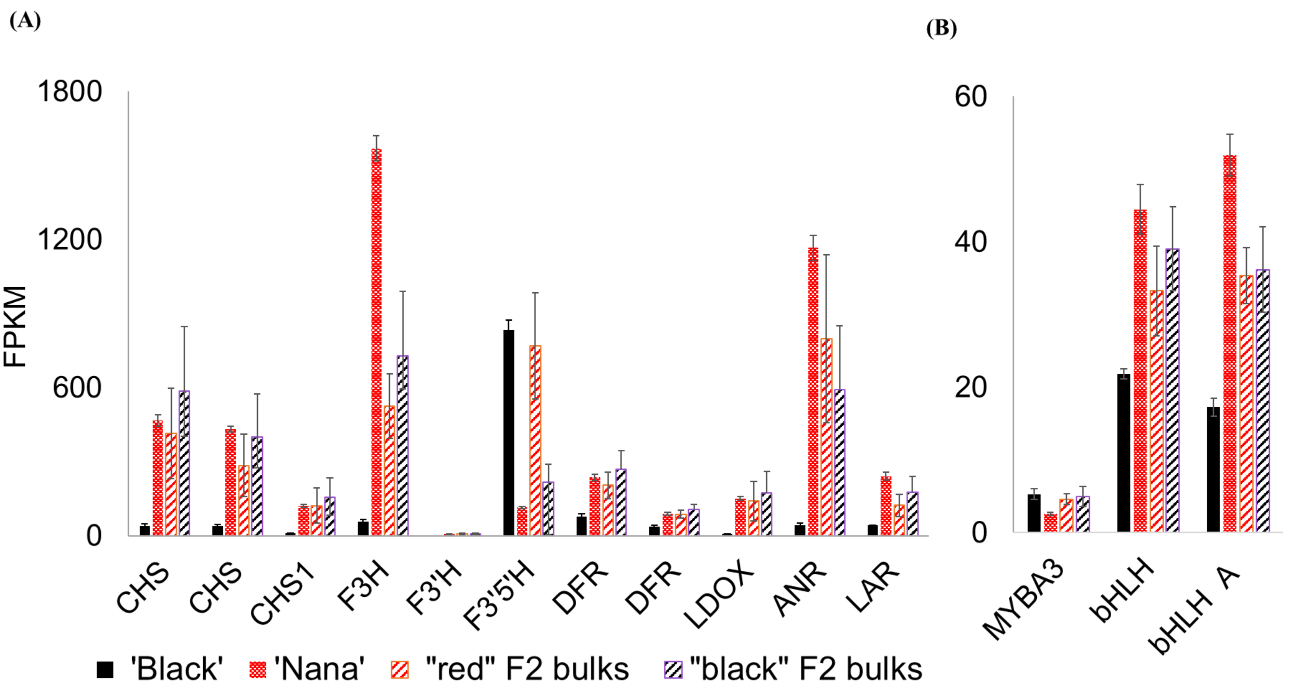

Supplementary Figure 1. Differential expression of genes from the anthocyanin pathway in young fruitlets of 'Black', 'Nana', and F_2 representative bulks ("red" and "black"). Peels were collected from fruits of 'Black' (black columns), 'Nana' (red columns), "red" F_2 bulks (red stripes) and "black" F_2 bulks (black stripes). Fruitlets were collected a few days after pollination. Expression analysis is explained in detail in the Materials and Methods section. **(A)** Genes corresponding to anthocyanin pathway, annotation is based on KEGG analysis. Gene names according to Qin et al., (2017): left to right: CDL15_Pgr005566 (CHS), CDL15_Pgr025723 (CHS), DL15_Pgr026373 (CHS1), CDL15_Pgr020918 (F3H), CDL15_Pgr008828 (F3'H), CDL15_Pgr026644 (F3'5'H), CDL15_Pgr021400 (DFR), CDL15_Pgr021400 (DFR), CDL15_Pgr017842 (LDOX), CDL15_Pgr017032 (ANR), CDL15_Pgr024128 (LAR). **(B)** Regulatory elements. From left to right- MYBA3 (JF747151), bHLH (CDL15_Pgr025), bHLH (JF747152). **(C)** Fruitlets analyzed showing the observed peel color at this stage. FPKM- Fragments per kilo base per million mapped reads). 'Nana' and 'Black' data presented are the same as in Figure 7.
