# Supplementary material for: Fine Mapping of the “black” Peel Color in Pomegranate (Punica granatum L.) Strongly Suggests That a Mutation in the Anthocyanidin Reductase (ANR) Gene Is Responsible for the Trait
Source: Front Plant Sci. 2021 Feb 25;12:642019. doi: 10.3389/fpls.2021.642019 (PMC7947214; doi:10.3389/fpls.2021.642019)
Supplement: Supplementary file 7 [file Table_6.DOCX]

Data of this project have been deposited with links to BioProject accession number PRJNA694423 in The National Center for Biotechnology Information (NCBI). BioSamples accession numbers : SAMN17522954, SAMN17523170 , SAMN17523467 , SAMN17523758  for 'Black', 'Nana', "red" peel bulks and "black" peel bulks, respectively. The Raw data from the Illumina sequencing have been deposited in NCBI Sequence Read Archive (SRA) under accession numbers:

| **accession** | **bioproject_accession** | **biosample_accession** | **library_ID** |
| --- | --- | --- | --- |
| SRR13530002 | PRJNA694423 | SAMN17522954 | Black rep1 |
| SRR13530001 | PRJNA694423 | SAMN17522954 | Black rep2 |
| SRR13529998 | PRJNA694423 | SAMN17522954 | Black rep3 |
| SRR13529997 | PRJNA694423 | SAMN17523170 | Nana rep1 |
| SRR13529996 | PRJNA694423 | SAMN17523170 | Nana rep2 |
| SRR13529995 | PRJNA694423 | SAMN17523170 | Nana rep3 |
| SRR13529994 | PRJNA694423 | SAMN17523467 | red peel bulks rep1 |
| SRR13529993 | PRJNA694423 | SAMN17523467 | red peel bulks rep2 |
| SRR13529992 | PRJNA694423 | SAMN17523467 | red peel bulks rep3 |
| SRR13529991 | PRJNA694423 | SAMN17523758 | black peel bulks rep1 |
| SRR13530000 | PRJNA694423 | SAMN17523758 | black peel bulks rep2 |
| SRR13529999 | PRJNA694423 | SAMN17523758 | black peel bulks rep3 |
